# How patch size and refuge availability change interaction strength and population dynamics: a combined individual- and population-based modeling experiment

**DOI:** 10.7717/peerj.2993

**Published:** 2017-02-21

**Authors:** Yuanheng Li, Ulrich Brose, Katrin Meyer, Björn C. Rall

**Affiliations:** 1 German Centre for Integrative Biodiversity Research (iDiv) Halle-Jena-Leipzig, Leipzig, Germany; 2 Institute of Ecology, Friedrich-Schiller Universität Jena, Jena, Germany; 3 Johann-Friedrich-Blumenbach Institute of Zoology and Anthropology, Georg-August-Universität Göttingen, Göttingen, Germany; 4 Department of Ecosystem Modelling, Georg-August-Universität Göttingen, Göttingen, Germany

**Keywords:** Functional response, Habitat loss, Habitat complexity, Food web, Individual-based model, Interaction strength, Population dynamics, Extinction, Patch size, Ordinary differential equation

## Abstract

Knowledge on how functional responses (a measurement of feeding interaction strength) are affected by patch size and habitat complexity (represented by refuge availability) is crucial for understanding food-web stability and subsequently biodiversity. Due to their laborious character, it is almost impossible to carry out systematic empirical experiments on functional responses across wide gradients of patch sizes and refuge availabilities. Here we overcame this issue by using an individual-based model (IBM) to simulate feeding experiments. The model is based on empirically measured traits such as body-mass dependent speed and capture success. We simulated these experiments in patches ranging from sizes of petri dishes to natural patches in the field. Moreover, we varied the refuge availability within the patch independently of patch size, allowing for independent analyses of both variables. The maximum feeding rate (the maximum number of prey a predator can consume in a given time frame) is independent of patch size and refuge availability, as it is the physiological upper limit of feeding rates. Moreover, the results of these simulations revealed that a type III functional response, which is known to have a stabilizing effect on population dynamics, fitted the data best. The half saturation density (the prey density where a predator consumes half of its maximum feeding rate) increased with refuge availability but was only marginally influenced by patch size. Subsequently, we investigated how patch size and refuge availability influenced stability and coexistence of predator-prey systems. Following common practice, we used an allometric scaled Rosenzweig–MacArthur predator-prey model based on results from our *in silico* IBM experiments. The results suggested that densities of both populations are nearly constant across the range of patch sizes simulated, resulting from the constant interaction strength across the patch sizes. However, constant densities with decreasing patch sizes mean a decrease of absolute number of individuals, consequently leading to extinction of predators in the smallest patches. Moreover, increasing refuge availabilities also allowed predator and prey to coexist by decreased interaction strengths. Our results underline the need for protecting large patches with high habitat complexity to sustain biodiversity.

## Introduction

The interplay between stability, complexity and biodiversity of ecological networks (especially food webs) is a subject of a long lasting and still ongoing discussion in ecology (e.g., MacArthur, 1955; May, 1972; McCann, 2000). From a mathematical point of view, an increase of species richness is only possible when the interaction strength between the species in a network (i.e., the strength of feeding interactions in a food web) decreases (May, 1972), otherwise species richness (represents biodiversity) must decline. A few possible solutions to overcome this problem have already been proposed, including a non-random organization of the feeding links in real food webs (Yodzis, 1981) and a decrease of specific interaction strength with increasing trophic levels (De Ruiter, Neutel & Moore, 1995), both driven by allometry (Yodzis & Innes, 1992; Brose, Williams & Martinez, 2006; Otto, Rall & Brose, 2007). Classic stability analyses assumed that the strength of interactions (the functional response) increased linearly with increasing resource availability (e.g., May, 1972). In food web studies, interaction strength refers to feeding interactions, which can be studied by investigating the ‘functional response’: how the number of prey eaten by a predator changes with increasing prey densities. In his seminal work, Holling (1959b) described the mechanism of functional responses mathematically and showed that under the simplest assumptions it should follow a hyperbolic curve. This experiment was performed in a simple experimental trial with blindfolded students (the predator) on a plain nine-square foot table as the experimental arena and sandpaper discs as prey. Interestingly, already in the same year Holling (1959a) showed that the shape of functional response could also follow a sigmoid (i.e., s-shaped) curve when investigating small mammals on a large natural scale, including natural complexity in the habitat. The different possible shapes of functional responses are known as type I functional responses (linear with a limit), type II functional responses (hyperbolic) and type III functional responses (sigmoid), forming the core set of functional responses alongside a number of other descendant types (Jeschke, Kopp & Tollrian, 2002). Notably, it has been shown that the type I functional response is an artifact (Jeschke, Kopp & Tollrian, 2004; Sarnelle & Wilson, 2008), so in this study we focus only on the non-linear functional responses. One of the descendants of Holling’s functional response models based on enzyme kinetics (Real, 1977) unifies the type II and the type III functional responses:

(1) \begin{eqnarray*}f(N)= \frac{{f}_{max}\,{N}^{h}}{{N}_{0}^{h}+{N}^{h}} \end{eqnarray*}

where *f*(*N*) is the per capita feeding rate, depending on the resource density, *N*. The curve is characterized by a maximum feeding rate, *f*_*max*_ [# h^−1^]; a half saturation density, *N*_0_ [# m^−2^], i.e., the prey density when the predator’s feeding rate reaches half of the maximum feeding rate and a unitless Hill exponent, *h*, determining the curve shape. If the Hill exponent is set to unity, the resulting function is the “strict” type II functional response. Whereas if the Hill exponent is set to two, it is the “strict” type III functional response in a very classical sense (but see Juliano (2001) for alternative descriptions of the type III functional response). We will subsequently refer to any functional responses as type III functional responses if the Hill exponent is larger than unity.

But why is it so important to know if the functional response is a type II functional response or a type III functional response? The answer is that type III functional responses are known to stabilize population dynamics thereby allowing for persistence of large food webs (Williams & Martinez, 2004; Brose, Williams & Martinez, 2006; Rall, Guill & Brose, 2008), by regulating prey populations to low densities (Nunney, 1980a; Nunney, 1980b). Several mechanisms have been put forward to explain why a type III functional response should appear, including the predator learning to exploit prey better (Holling, 1966) and switching between different prey types to the most abundant prey (Murdoch & Oaten, 1975; Oaten & Murdoch, 1975). More recently, it was suggested that refuges for the prey can also lead to a type III functional response (e.g., Scheffer & De Boer, 1995; Vucic-Pestic et al., 2010a). However, there was only mixed support from studies manipulating habitat complexity in general without introducing prey refuges explicitly (Kaiser, 1983; Hoddle, 2003; Hohberg & Traunspurger, 2005; Hauzy et al., 2010; Vucic-Pestic et al., 2010a; Kalinkat, Brose & Rall, 2013). Those differences may be caused by variations in how habitat complexity influences foraging and hence feeding: (1) complexity negatively affects feeding by e.g., refuges restraining predation especially at low prey densities and eventually leading to a type III functional response; (2) it affects feeding by e.g., obstacles preventing the movements of both predator and prey leading to reduced encounter rates at all prey densities but leaving the functional response type unaffected (Hauzy et al., 2010). Moreover, if the habitat complexity influences neither movement nor refuge provision, it will simply cause a dilution effect, a virtual increase of the patch size (Kalinkat, Brose & Rall, 2013).

Furthermore, functional response studies are predominantly carried out under artificial laboratory conditions (Jeschke, Kopp & Tollrian, 2004; Kalinkat & Rall, 2015). This means that (1) most of the studies mentioned above have used rather artificial habitat complexity and (2) due to spatial limitations of a laboratory, the size of the experimental units are relatively small (e.g., petri-dishes for estimating the functional response of ladybugs, *Stethorus japonicus* (Gotoh, Nozawa & Yamaguchi, 2004)). Only a few studies have attempted to investigate functional responses in natural environments, but these studies are only roughly comparable to the controlled laboratory studies as they rely on scat counting or gut content analyses combined with assessments of natural prey density  (e.g., Dale, Adams & Bowyer, 1994; Smout & Lindstrøm, 2007). To our knowledge, only one study so far has investigated and compared the simplified laboratory functional response experiments (using petri dishes) with functional responses measured in the greenhouse or in the field (Munyaneza & Obrycki, 1997). In this study, the attack rates in the laboratory were over 40 times higher than those in the greenhouse but those in the field were three to nine times lower than in the petri dishes (note that all functional responses in the original publication were fitted to a type II functional response model and the attack rates were scaled to the total size of the experimental arena, we compared the attack rates scaled to square meters (Rall et al., 2012)). As the experiments carried out by Munyaneza & Obrycki (1997) altered habitat complexity and patch size at the same time, and both gradients increased simultaneously from the petri dish experiments over the greenhouse to the field experiments, it was not possible to disentangle the potential interactive effect of habitat complexity and patch size. Furthermore, Bergström & Englund (2004) reported increases in attack rates with patch size, and studies manipulating habitat complexity reported a decrease in attack rates (e.g., Vucic-Pestic et al., 2010a). This might lead to the explanation that the relatively low attack rates in the field shown in the study of Munyaneza & Obrycki were due to the increased patch size and habitat complexity.

Beside the examples above, we are not aware of any other study addressing the effect of patch size and habitat complexity on the functional-response parameters. Moreover, most of the studies only vary habitat complexity or patch size by up to four levels (e.g., Kalinkat, Brose & Rall, 2013; Bergström & Englund, 2004) and none of them systematically varied both complexity and size. This lack of studies is perhaps due to the laborious nature of functional response studies. For example, Vucic-Pestic et al. (2010b) used prey ranging from one up to 4,000 individuals for fitting a single functional response, measuring up to 90 feeding experiments. Doubling the patch size would already lead to a maximum of 8,000 individuals and a 10 times larger patch would require already a maximum of 40,000 individuals.

As such extreme laboratory settings are not feasible, we developed an individual-based model (IBM) to study the effects of patch size and refuge availability on functional-response parameters. We explored full-factorial patch size and habitat complexity to disentangle effects of both variables and eventually their interactive effects on the functional-response parameters. Subsequently, we analyzed the stability of a predator-prey system depending on patch size and habitat complexity by developing a predator-prey population dynamics model which has a long standing usage and wide applicability (see section “Methods” for details).

## Methods

### Individual-based model of feeding interaction

#### Overview

To investigate the effects of patch size and habitat complexity (represented by refuge availability) on functional-response parameters, we developed an individual-based allometric predator-prey model (for details, see the supplement for an **O**verview, **D**esign concepts, **D**etail protocol, Grimm et al. (2006); Grimm et al. (2010)) to mimic the feeding experiments in the laboratory. We assumed that the maximum feeding rate was driven by mechanical and physiological processes such as chewing and digestion and would not scale with patch size or refuge availability. Therefore, we first investigated the maximum feeding rate without any explicit space properties. Second, we modeled a two-dimensional square area to mimic an explicit patch in which both predator and prey can continuously move. The modeled patch consisted of cells all individuals can enter; however, cells may be marked as refuges preventing predation.

#### The model processes

**Figure 1:**
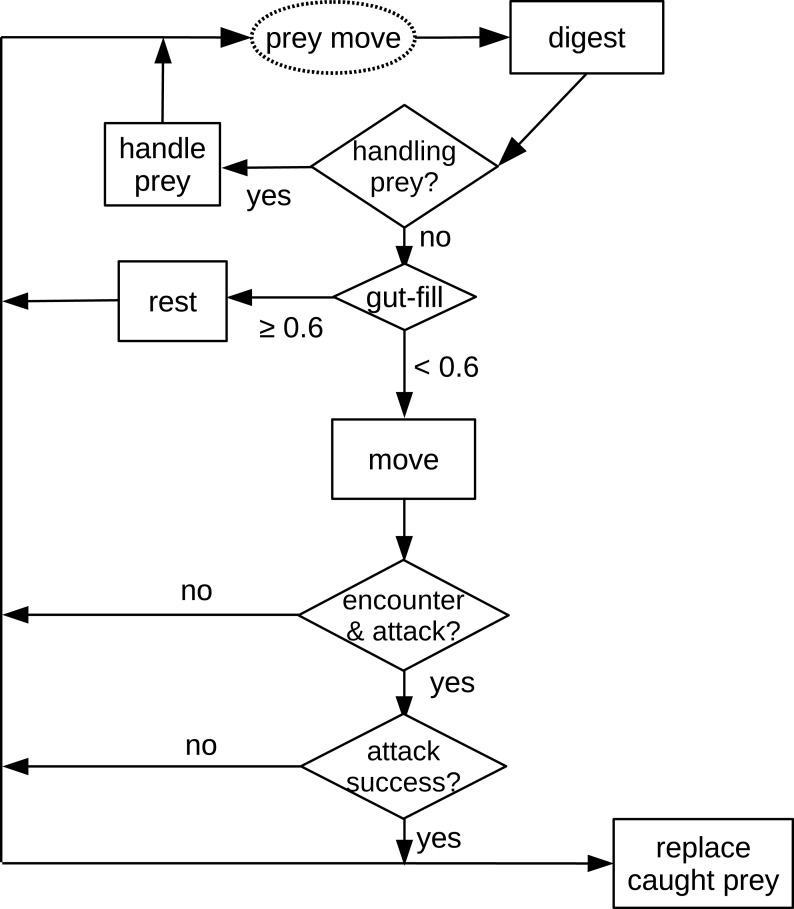
Schematic diagram of processes in the *in silico* feeding experiment model. The text in the dashed oval is the only prey action which is also the start of the processes. Texts in the squares are decisions or actions of the predator. Texts in the diamonds are decisions making, e.g., “handling prey?”

The first process applied in the model is prey movement (random walk with randomly chosen direction, 0–2*π* double precision floating number and allometrically calculated distance). The following processes applied in the model are all decisions and actions of the predator (Fig. 1). First, the digestion of the predator is calculated. Subsequently, the algorithm checks if the predator is handling prey (caught in an earlier time step). If not, and the predator’s gut is full (≥ 60%), it rests (not taking further actions). If the predator is not handling prey and is hungry (gut filling < 60%), the predator moves (random walk, see above). After reaching the new position, the predator investigates if it encounters a prey in the cell. If there is a prey individual in the same cell, it will be attacked. If the attack is successful, another prey item is placed randomly into the grid to keep the prey density constant. The predator starts to handle (chew) prey in the next time step.

#### Variables and parameters

Most species traits regulating the processes described above follow allometric rules (Kleiber, 1961; Peters, 1983; Brown et al., 2004; Brose, 2010), including velocity, *V* [cm s^−1^], of both the predator and the prey (Peters, 1983); and the traits of the predator: gut size, *G* [mg] (Ibarrola et al., 2012), digestion rate, *D* [mg s^−1^] (Ibarrola et al., 2012), handling time, *T*_*h*_ [s] (estimated from Rall et al., 2012, see Supplemental Information) and attack success, *S*_*a*_ [unitless] (Brose et al., 2008; Gergs, 2011):

(2a) \begin{eqnarray*}V={v}_{0}\,{M}^{{a}_{v}}\end{eqnarray*}

(2b) \begin{eqnarray*}G={g}_{0}\,{M}^{{a}_{g}}\end{eqnarray*}

(2c) \begin{eqnarray*}D={d}_{0}\,{M}^{{a}_{d}}\end{eqnarray*}

(2d) \begin{eqnarray*}{T}_{h}={h}_{0}\,{M}_{p}^{{a}_{h,p}}\,{M}_{n}^{{a}_{h,n}}\end{eqnarray*}

(2e) \begin{eqnarray*}{S}_{a}={a}_{0}\,{ \left( \frac{R}{{R}_{opt}} \,{e}^{1- \frac{R}{{R}_{opt}} } \right) }^{\lambda }\end{eqnarray*}

where *v*_0_, *g*_0_, *d*_0_ and *h*_0_ are constants, *a*_*v*_, *a*_*g*_, *a*_*d*_ and *a*_*h*_ are the allometric scalings, and *M* is the body mass of the corresponding individual. Subscripts, _*p*_ and _*n*_ indicate predator and prey respectively. We used the widespread generalized Ricker’s function (Persson et al., 1998; Persson & Brönmark, 2002b; Persson & Brönmark, 2002a; Wahlström et al., 2000; Brose et al., 2008; Rall et al., 2011) to describe the scaling of attack success depending on body mass. This function consists of the maximum attack success *a*_0_, predator-prey body-mass ratio, *R* and its optimum *R*_*opt*_ and a shaping parameter, *λ*. Predator and prey also possessed some state variables to assist their decision making and activities, i.e., the ‘position’ for all individuals; the ‘gut fullness’ and if the predator is ‘still handling’ and an identifier, ‘prey identity,’ to distinguish between the prey individuals.

#### Parameters’ range

**Figure 2:**
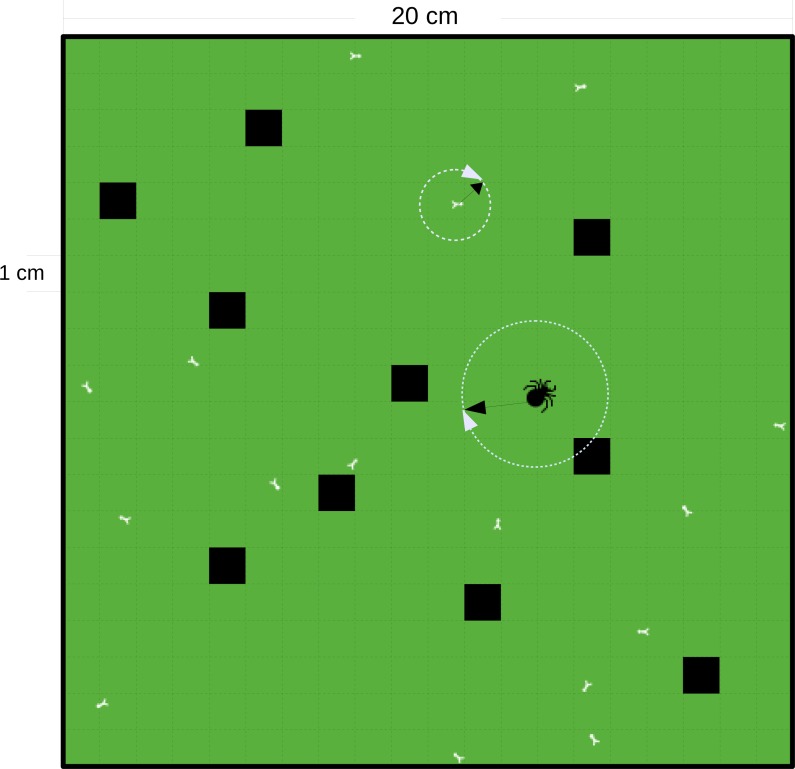
Schematic diagram of a square grid of the IBM model. The grid in this example is 0.04 m^2^ with cell resolution of 1 cm × 1 cm and with a ‘wall-boundary condition’ (individuals not able to penetrate the walls). The black cells are the refuge cells. The small white bugs represent prey and the big black bug represents the predator. The random walk of the individuals is decided by a randomly chosen direction, 0–2*π* and an allometrically decided distance (as denoted by the white circles and black arrows).

The cell resolution of the square grid, in which the *in silico* simulations are conducted is 1 cm × 1 cm. As we intended to mimic laboratory experiments, the walls of the grid are set to ‘wall-boundary condition’ (individuals cannot penetrate the walls). We chose twelve patch sizes ranging from 0.2 m × 0.2 m = 0.04 m^2^ (the size of a standard patch in some terrestrial functional response experiments (Brose et al., 2008; Rall et al., 2010; Vucic-Pestic et al., 2010a; Rall et al., 2011; Vucic-Pestic et al., 2011; Kalinkat, Brose & Rall, 2013) to 100 m^2^ (the size of a field patch (Munyaneza & Obrycki, 1997)). The sizes of each patch were: 0.04 m^2^, 0.16 m^2^, 0.64 m^2^, 1.44 m^2^, 2.56 m^2^, 4 m^2^, 16 m^2^, 36 m^2^, 49 m^2^, 64 m^2^, 81 m^2^, and 100 m^2^. The second independent variable we modeled was prey refuge that served as a surrogate for habitat complexity which preventing feeding. We randomly selected refuge cells on the grid for each simulation in a certain percentage of cells in steps of 5% (5%–75% as the ratio of refuge cells to all cells); see Fig. 2 as a case example. These two independent variables are full-factorially simulated. For each simulation run, the refuge distribution is newly drawn. Those randomly chosen cells do not support any feeding by the predator and therefore act as refuges for the prey. The body masses of the predator and prey were set to 100 mg and 1 mg, a common body-mass ratio for animal predatory interactions, close to the optimal feeding ratio of invertebrates (e.g., Vucic-Pestic et al., 2010b; Rall et al., 2011; Kalinkat et al., 2013). We ran each of the *in silico* feeding trials for 3,600 steps (representing 1 h). The simulation for estimating the maximum feeding rate was repeated 50 times and each prey density dependent simulation was repeated five times. We simulated prey densities from 2^0^ to 2^*n*^ as the density when the predator (only one predator per simulation) is satiated. For example, twenty prey densities from 2^0^ to 2^19^ are selected for the patch size of 36 m^2^ and 35% refuge-area ratio. Values for the parameters in allometric equations, Eq. (2), are empirically-based and given in Table 1. These values (Table 1) are derived from the same studies where we derived the formulas. Yet the maximum attack success *a*_0_ is taken as the mean of 5 measurements from Gergs (2011). The optimum predator-prey body-mass ratio is consistent with terrestrial invertebrates from Brose et al. (2008).

**Table 1:** Parameters values in allometric equations (Eq. (2)).

Parameter	Value	Parameter	Value
*v*_0_	0.546	*a*_*v*_	0.29
*g*_0_	0.50	*a*_*g*_	0.434
*d*_0_	5 × 10^−5^	*a*_*d*_	0.75
*h*_0_	37.504	*a*_*h*,*p*_	−0.330
		*a*_*h*,*n*_	0.173
*a*_0_	0.10	*R*_*opt*_	100
		*λ*	1

### Statistics

#### Functional response fitting

We first calculated the mean maximum feeding rate for the predator-prey pair. We used a generalized linear model (GLM) assuming that maximum feeding rates follow Poisson distribution as feeding rates were count data of non-negative integers of which the error distribution increases with increasing mean. The statistics were ran in *R* (R Core Team, 2016), but see chapter 13 in Crawley (2007) for details. Subsequently, we used this mean maximum feeding rate as a fixed parameter in the functional response model (Eq. (1)) to estimate the dependencies of the remaining half saturation density and Hill exponent.

We analyzed the feeding data from IBM models using Real’s functional response, Eq. (1). As there is no well-established scaling relationships of functional-response parameters (half saturation density and Hill exponent) to habitat properties investigated here, i.e., patch size and refuge availability, we preliminarily tested whether the scalings of these functional-response parameters followed a power law or exponential function. To reduce the potential influences of interaction terms (between patch size and refuge availability) which may influence the dependencies of the half saturation density or Hill exponent, we included all interaction terms in the preliminary testing (Zuur et al., 2009). We analyzed in total 16 full models and compared them using the Bayesian Information Criterion (BIC), see Table S3. This analysis revealed that the scalings of half saturation densities with patch size and refuge availability can be best described by a power law and an exponential function, respectively:

(3) \begin{eqnarray*}{N}_{0}={C}_{{N}_{0}} {A}^{{a}_{{N}_{0}}} {e}^{{b}_{{N}_{0}}\,R} {e}^{{\gamma }_{{N}_{0}}\,\ln \nolimits \left( A \right) \,R}\,\end{eqnarray*}

where *C*_*N*_0__ is a constant, *a*_*N*_0__ is the scaling exponent of half saturation density to patch size, *A*, *b*_*N*_0__ is the scaling parameter of half saturation density to refuge availability, *R* and *γ*_*N*_0__ is the parameter giving the strength of the interaction between patch size and refuge availability. Preliminary analyses also showed that the Hill exponent depended on patch size and refuge availability both following power laws:

(4) \begin{eqnarray*}h={C}_{h} {A}^{{a}_{h}} {R}^{{b}_{h}} {e}^{{\gamma }_{h}\,\ln \nolimits \left( A \right) \,\ln \nolimits \left( R \right) }\,\end{eqnarray*}

where *C*_*h*_ is a constant, *a*_*h*_ is the scaling parameter of the Hill exponent to patch size, *A*, *b*_*h*_ is the scaling exponent of the Hill exponent to refuge availability, *R* and *γ*_*h*_ is the parameter giving the strength of the interaction between patch size and refuge availability.

We fitted the functional response model, Eq. (1) with the dependencies described above using a maximum likelihood method, ‘mle2()’ (Bolker & R Development Core Team, 2014), (see Bolker (2008) for details). As we replaced eaten prey after each feeding event (see above), we assumed that the residuals followed a negative binomial distribution. We fitted this functional response model to the data assuming a log link between data and model:

(5a) \begin{eqnarray*}\ln \nolimits \left( {N}_{0} \right) =\ln \nolimits \left( {C}_{{N}_{0}} \right) +~{a}_{{N}_{0}}\,\ln \nolimits \left( A \right) +~{b}_{{N}_{0}}\,R +~{\gamma }_{{N}_{0}}\ln \nolimits \left( A \right) \,R\,\end{eqnarray*}

(5b) \begin{eqnarray*}\ln \nolimits \left( h \right) =\ln \nolimits \left( {C}_{h} \right) +~{a}_{h}\,\ln \nolimits \left( A \right) +~{b}_{h}\,\ln \nolimits \left( R \right) +~{\gamma }_{h}\,\ln \nolimits \left( A \right) \,\ln \nolimits \left( R \right) \,\end{eqnarray*}

i.e., we did not fit the values for the constants *C* in Eqs. (3) and (4), but for the intercepts in the ln-transformed version ln(*C*_*N*_0__) and ln(*C*_*h*_) in Eq. (5). We performed a model selection using the Bayesian Information Criterion (BIC) by comparing all possible combinations of setting the parameters *a*, *b* and *γ* to “0,” resulting in 25 meaningful combinations (note that either *a* or *b* only can be excluded if the interaction term, *γ* is excluded).

### Population dynamics model

To investigate how patch size, *A*, and refuge availability (a measurement of habitat complexity), *R*, affect population dynamics and stability of a predator-prey system, we set up an ordinary differential equations (ODE) model. Such models were widely used to study one population (e.g., Gompertz, 1825; Verhulst, 1838) over food web motifs (e.g., Lotka, 1925; Volterra, 1926; Rosenzweig & MacArthur, 1963; Rosenzweig, 1971; Yodzis & Innes, 1992) to multi-trophic food web models (e.g., Williams & Martinez, 2004; Binzer et al., 2016; Schneider et al., 2016) and used to predict patterns of experimental microcosms (e.g., Schneider, Scheu & Brose, 2012; Fussmann et al., 2014) up to whole food webs (e.g., Boit et al., 2012). The ODE model describes the change in prey density [# m^−2^], *dN*, and predator density [# m^−2^], *dP*, over time, *dt* (Rosenzweig & MacArthur, 1963; Yodzis & Innes, 1992; Otto, Rall & Brose, 2007):

(6) \begin{eqnarray*} \frac{dN}{dt} =rN \left( 1- \frac{N}{K} \right) -\omega \, \frac{{f}_{max}\,{N}^{h}}{{N}_{0}^{h}+{N}^{h}} \,P\,\end{eqnarray*}

(7) \begin{eqnarray*} \frac{dP}{dt} =e\,\omega \frac{{f}_{max}\,{N}^{h}}{{N}_{0}^{h}+{N}^{h}} \,P-xP\end{eqnarray*}

where the prey growth is described by logistic growth with *r* [d^−1^] being the intrinsic growth rate and *K* [# m^−2^] being the carrying capacity. The prey are consumed by the predator following Real’s functional response, Eq. (1), with *f*_*max*_ being the maximum feeding rate, *N*_0_ being the half saturation density and *h* being the Hill exponent. The predator population grows according to the functional response multiplied by the assimilation efficiency, *e*, and the effective foraging time proportion *ω*. Moreover, it loses population density by metabolism, *x* [d^−1^].

We used the estimated values from the functional response fitting of our individual-based model (see above) in the ODEs. Additionally we calculated the values for carrying capacity, *K*, growth rate, *r* and metabolism, *x*, according to empirically derived studies (Rall et al., 2010; Meehan, 2006; Savage et al., 2004a; Peters, 1983) (details described afterwards).

#### Equilibrium densities of the predator-prey system and extinction boundaries

The predator-prey population model has a set of non-trivial analytical solutions, being a predator isocline

(8) \begin{eqnarray*}N={ \left( \frac{x\,{{N}_{0}}^{h}}{e\,\omega \;{f}_{max}-x} \right) }^{ \frac{1}{h} }\end{eqnarray*}

and a prey isocline

(9) \begin{eqnarray*}P=r\,{N}^{1-h}(k-N)\, \frac{{{N}_{0}}^{h}+{N}^{h}}{k\,\omega \;{f}_{max}} \,.\end{eqnarray*}

After obtaining these isoclines, the equilibrium densities of predator and prey are compared with extinction boundaries [# m^−2^]. Such boundary is set to an artificial small number in the common practice of ODE models, but we explicitly set it to two individuals per patch. In cases where the predator population is not sustained, i.e., the equilibrium density is less than the extinction boundary, prey population would grow to its capacity, *N* = *K* and the predator population goes extinct, *P* = 0.

#### Parameter values for the ODE

The functional-response parameters, the maximum feeding rate, *f*_*max*_, the half saturation density, *N*_0_ and the Hill exponent, *h*, are set according to the statistical results of the IBM simulations. We assumed that the predator foraged approximately 12 h a day (Ebeling & Bray, 1976), therefore we added a foraging time proportion $\omega = \frac{1}{2} $. The assimilation efficiency, *e*, accounts for the proportion of food overwhelmed by the predator which can be converted to its own body mass, which is set to 0.85, a common value for predatory consumers (Yodzis & Innes, 1992; Otto, Rall & Brose, 2007). The prey growth follows the logistic growth consisting of the intrinsic growth rate *r* and the carrying capacity *K*. Together with metabolic rate of the predator, these three parameters are calculated by empirically derived equations.

(10a) \begin{eqnarray*}K={K}_{0}\,{M}_{n}^{{b}_{K}}\,{e}^{ \frac{{E}_{K}}{k\,T} }\,({\sigma }_{0}\,{e}^{ \frac{{E}_{\sigma }\,({T}_{0}-T)}{k\,T\,{T}_{0}} })^{z}\,{e}^{{tl}_{0}\,(tl-1)}\,\end{eqnarray*}

(10b) \begin{eqnarray*}r={r}_{0}\,{M}_{n}^{{b}_{r}}\,{e}^{ \frac{{E}_{r}}{k\,T} }\end{eqnarray*}

(10c) \begin{eqnarray*}x=\sigma \,{c}_{x}\,{x}_{0}\,{M}_{p}^{{b}_{x}}\,{e}^{ \frac{-{E}_{x}}{k\,T} }\end{eqnarray*}

The carrying capacity *K* scales with body mass, *M*_*n*_ (gram), environmental temperature, *T* (K), net primary production of the habitat, $({\sigma }_{0}{e}^{ \frac{{E}_{\sigma }({T}_{0}-T)}{kT{T}_{0}} })^{z}$, and the trophic level of the prey, *tl*. The values for all parameters are derived for invertebrate detritivores assuming German weather conditions and productivity: *K*_0_ = *e*^−31.15^; *b*_*K*_ =  − 0.72; *E*_*K*_ = 0.71; *k* = 8.62*e* − 05; *T* = 282.65; *σ*_0_ = 600; *E*_*σ*_ =  − 0.35; *T*_0_ = 293.15; *z* = 1.03; *tl*_0_ =  − 2.68; *tl* = 1.5 (see Meehan (2006) and Rall et al. (2010) for details). The growth rate *r*, scales with body mass (microgram) and environmental temperature, where *r*_0_ = *e*^32.39^, *b*_*r*_ =  − 0.25 and *E*_*r*_ =  − 0.84 (details see Savage et al. (2004a) and Rall et al. (2010)). The metabolic rate *x*, also scales with body mass (gram) and environmental temperature with *x*_0_ = *e*^27.68^, *b*_*x*_ = 0.72 and *E*_*x*_ = 0.87 (see Peters (1983); Savage et al. (2004b) and Rall et al. (2010) for details). Savage et al. (2004b) reported that field metabolic rate were three times larger than basal, therefore we include the coefficient *σ* as 3. The normalization constant *c*_*x*_, $12342.86 {M}_{p}^{-1}$ (*M*_*p*_ in milligram), converts the metabolism from J s^−1^ to d^−1^ (Peters, 1983).

We set predators to 100 mg, and prey to 1 mg, consistent with our individual-based model simulations described above. We also explored the same ranges of patch size and habitat complexity as for the individual-based model simulations explained above. Extinction boundaries for predator and prey were set to two individuals per patch.

## Results

### Results of IBM simulation

**Figure 3:**
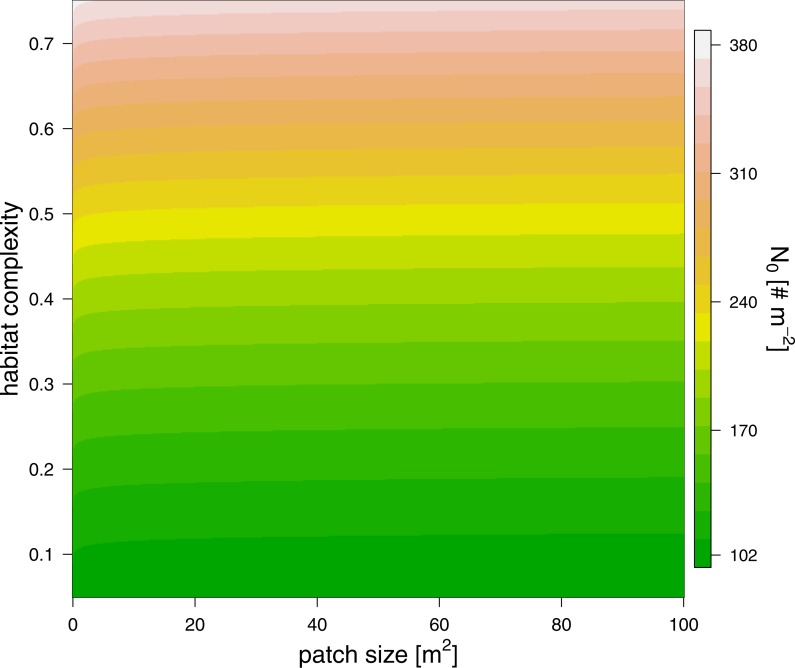
The effect of patch size (*x*-axis) and refuge availability (*y*-axis) on half saturation density (see color scale).

**Figure 4:**
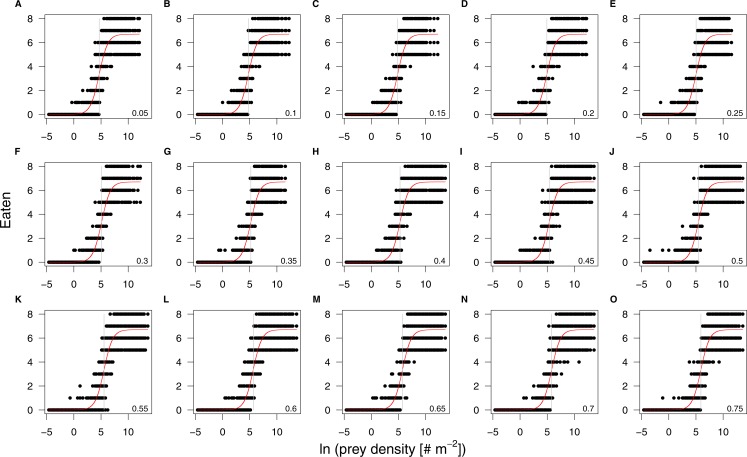
Results of the individual-based functional response *in silico* experiments (black dots) and their corresponding fits (red lines). The panels are arranged by increasing refuge availability, starting at 5% (A) to 75% (O). The patch size effect on the half saturation density is too small to result in visually distinguishable regression lines. All prey densities are ln-transformed. The grey vertical lines denote the half saturation densities.

The maximum feeding rate, *f*_*max*_, was estimated prior to the functional response fitting and revealed that predators of 100 mg fed in average 6.7 prey individuals per hour (Table 2, note that the statistics were performed using a log-link function, i.e., the ln-linear feeding rate was estimated). We subsequently fitted the functional response with a fixed maximum feeding rate. Our model comparison of patch size and refuge availability dependent functional response models, based on BIC, included a scaling of half saturation density with patch size and refuge availability, but it did not include any scaling of the Hill exponent with either parameter (Table 2). The half saturation density increased with refuge availability, and decreased marginally with patch size, see Fig. 3. The estimated Hill exponent across patch sizes and refuge availabilities was 1.284, which is significantly different from a Hill exponent of 1 therefore indicating a type III functional response (Table 2, note that the ln-transformed Hill exponent was tested against “0” what is a Hill exponent of “1”). The emerging functional responses are of the same shape but feeding is realized at higher prey densities with increasing refuge availability (Fig. 4). Other predator-prey body-mass ratios showed similar results, see the section “*In silico* feeding experiments on other body-mass ratios” in the supplement.

**Table 2:** Statistical results for the *in silico* functional response experiments. Note that the maximum feeding rate is a priori assumed to be independent of patch size and refuge availability.

		Estimate	S.E.	*p*-value
Maximum feeding rate	ln(*f*_*max*_)	1.902	0.05	<0.001
Half saturation density	ln(*C*_*N*_0__)	4.577	0.031	< 0.001
	*a*_*N*_0__	−0.007	0.005	0.21
	*b*_*N*_0__	1.777	0.063	< 0.001
	*γ*_*N*_0__	Excluded by model selection
Hill exponent	ln(*C*_*h*_)	0.25	0.011	< 0.001
	*a*_*h*_	Excluded by model selection
	*b*_*h*_	Excluded by model selection
	*γ*_*h*_	Excluded by model selection

### Results of population dynamic model

**Figure 5:**
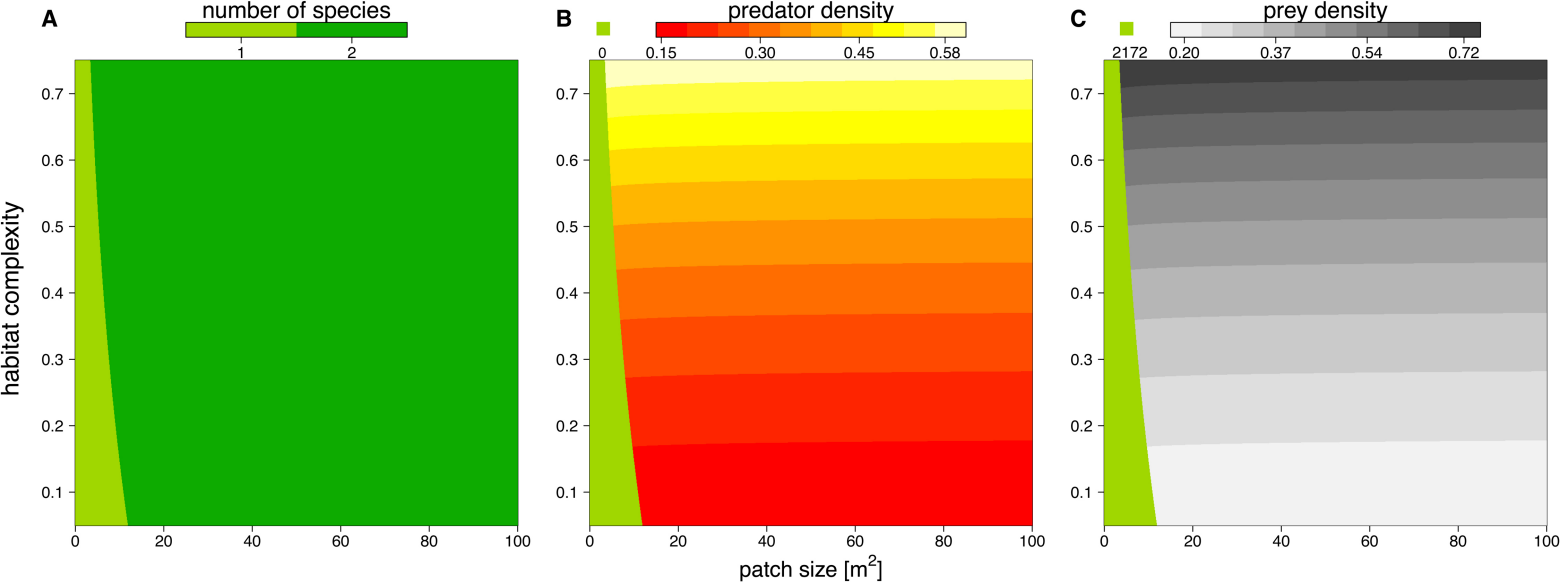
Number of surviving species (A), population densities of predator (B) and prey (C) depending on patch size (*x*-axis) and refuge availability (*y*-axis). When the system is embedded in very small patches, the predator becomes extinct due to energy limitation and the prey grows to its carrying capacity (green areas in B and C). The non-green areas of B and C show the densities, [# m^−2^], of predator and prey when the system is feasible (see color code above each plot).

We solved the population dynamics model by a set of analytical solutions (Eqs. (8) and (9)) and the extinction boundaries. In small patches only the prey species survived, but refuge availability relaxed this pattern, allowing predators to survive at smaller patches. Both predator and prey population densities increased with refuge availability, whereas in larger patches, the densities of predator and prey populations decreased slightly (Fig. 5).

## Discussion

### Effects of spatial properties on interaction strength

We developed an allometric individual-based model to investigate the effects of patch size and habitat complexity (represented by refuge availability) on feeding interactions. We found that the interaction strength decreased with refuge availability, as the half saturation density increased with it. This result is consistent with most of empirical studies aiming to account for how refuges affect predation rates (e.g., Kaiser, 1983; Folsom & Collins, 1984; Kalinkat, Brose & Rall, 2013). Our results showed that patch size, however, did not significantly influence the interaction strength. Bergström & Englund (2004) reported that the attack rate (the maximum interaction strength at low prey densities) increased with patch size. This increase was explained by behavioral changes in moving activity (increasing speed of the predator) and an aggregative behavior of both their prey and predator at the walls of their experimental aquariums (animals clustered more at the aquarium walls with increasing patch size). We did not include such behavioral changes in our model as we wanted to provide a simple basic model in this study, which may explain the differences of our results from Bergström & Englund (2004). The Hill exponents in our *in silico* individual-based experiments were 1.28 across patch sizes and refuge availabilities. This is quite surprising, as a simple type II functional response was thought to be the appropriate model for feeding interaction experiments under simplified conditions in the laboratory. However, the empirical findings of Sarnelle & Wilson (2008) suggested that type III functional responses would emerge if researchers were able to include experimental trials on small prey densities, which was not feasible for experiments carried out in small patches. A few feeding interaction studies on mammals (‘intelligent predators’) carried out in the field also suggested type III functional responses (Holling, 1959a; Smout & Lindstrøm, 2007). More recent studies found type III functional responses for invertebrates as well (Aljetlawi, Sparrevik & Leonardsson, 2004; Vucic-Pestic et al., 2010b). Our study not only corroborates the finding of type III functional response, but also confirms that for the mechanistically simplified predators as in our individual-based model, a type III functional response is appropriate, which is not only suitable for ‘intelligent predators’ with the ability to learn (Holling, 1966). The statistical results for *in silico* experiments of other body-mass ratios showed consistency with the results discussed above.

Former laboratory experiments that compared a homogeneous habitat with a complex habitat documented a shift from a type II to a type III functional response (Vucic-Pestic et al., 2010a) and argued that this was due to a refuge effect. We did, however, not find any increase in the Hill exponent with increasing refuge availability. As we did not include explicit behaviors for hiding, we infer that this switch from a type II to a type III functional response (or an increase of the Hill exponent) not only needed refuges as shelter for the prey, but also active behavioral changes in sub-habitat choice (Schmitz, Krivan & Ovadia, 2004; Miller, Ament & Schmitz, 2014).

Our individual-based predator-prey model framework allowed us to investigate the effects of patch size and refuge availability on functional-response parameters, which would not have been possible in laboratory or field experiments. Even without incorporating more complex movement models than random walks or behaviors like chasing or hiding, we were able to detect general patterns on the scalings of functional-response parameters with increasing patch size and refuge availability. Nevertheless, future individual based predator-prey models should incorporate more complex movement models to better understand the mechanisms of functional responses.

### Effects of spatial properties on population dynamics

**Figure 6:**
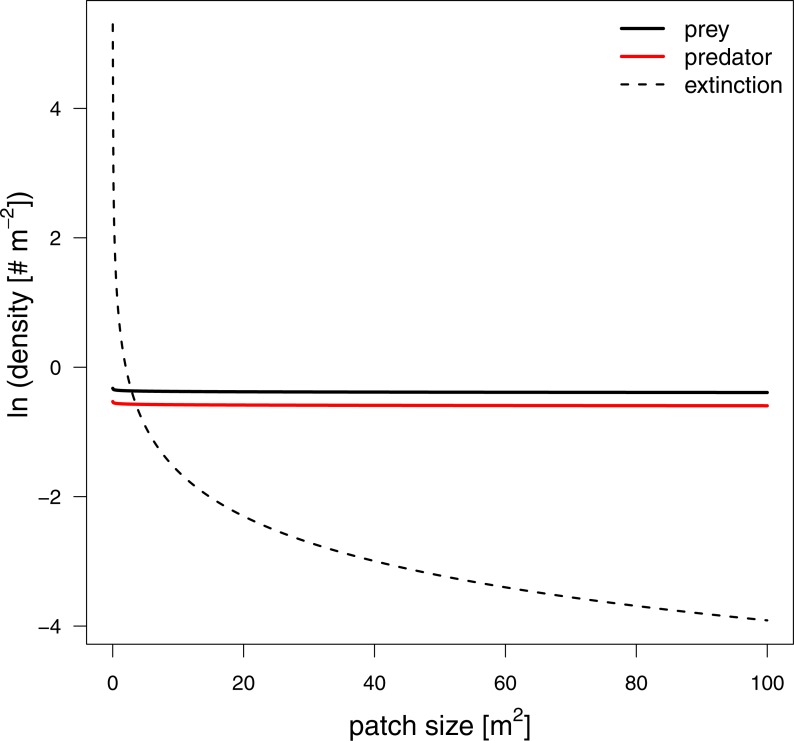
Equilibrium densities and extinction boundaries of the predator-prey system. This is an example where 73% of the cells are prey refuges. The solid lines depict the equilibrium population densities of the predator (red) and prey. The dashed line indicates the extinction boundaries of the predator and prey, two individuals per patch.

To investigate how changes in interaction strength scale up to population dynamics and coexistence, we analyzed a predator-prey ordinary differential equation model. We used the results from our *in silico* feeding experiments and combined it with empirically measured values for growth, carrying capacity and metabolism (Meehan, 2006; Savage et al., 2004a; Savage et al., 2004b; Brown et al., 2004; Rall et al., 2010). Increasing patch size turned the extinction of predators to survival, meaning the smallest patches were not able to sustain the predator population. This is surprising as the feeding interaction strength does not change with patch size (i.e., a non-significant effect of patch size). As all parameters of the model are constant in respect to patch size, we expected that neither the stability (*sensu* population dynamics) nor persistence will be affected. This paradox behavior of the system can only be explained by the increasing extinction thresholds with decreasing patch size. We defined the extinction threshold as two individuals per patch leading to increasing densities for extinctions with decreasing patch size (see Fig. 6 as an example). Increasing refuge availability counteracted this pattern and allowed predators to survive at even smaller patches. This is surprising as the half saturation density increased with refuge availability, suggesting less energy intake by the predator. However, increasing half saturation density also led to an increase in prey density in equilibrium that subsequently sustained a higher predator density. Both predator and prey have been feasible in larger patches and across the range of habitat complexities we explored.

Using a predator-prey population dynamics model, parameterized by the *in silico* functional response experiments discussed above, we were able to detect patterns of coexistence when patch size and refuge availability increased. These results are predominantly driven by the incorporation of a nearly constant feeding interactions with increasing patch size and a realistic assumption for the extinction boundaries of populations which is usually ignored in an ordinary differential equation modeling frameworks (e.g., McCann, Rasmussen & Umbanhowar, 2005; Otto, Rall & Brose, 2007). Future studies that aim to investigate effects of space on persistence using ordinary differential equation models should consider to include such more realistic extinction boundaries as presented in our study.

## Conclusions

How species interactions react to environmental changes such as habitat homogenization and habitat loss is a key point for understanding how current global changes (IPCC, 2014) influence the stability and biodiversity of ecological networks. Increasing the stability of food webs is possible via obtaining weaker interaction strengths (May, 1972) which is important for maintaining biodiversity. We found that loss of habitat complexity would lead to increased interaction strength via decreasing half-saturation density. Additionally, even though the constant interaction strengths through different patch sizes lead to constant population densities, it would result in a less absolute number of individuals in smaller patches. When there are a reduced number of individuals in smaller patches, decreasing patch size would cause species extinctions, especially at higher trophic levels. Therefore, shrinking patch sizes and homogenizing habitats would both lead to destabilization of ecological networks and biodiversity loss. Altogether, our study underlines the urgent need for protecting large complex habitats to save biodiversity.

##  Supplemental Information

10.7717/peerj.2993/supp-1 Supplemental Information 1 CPP code for the individual based model Includes a header file, a functions file and a main file to reproduce the model described in the main text.

10.7717/peerj.2993/supp-2 Supplemental Information 2 Supplement Contains the ODD and further information about parameters.

## Additional Information and Declarations

### Data Availability

The following information was supplied regarding data availability: The raw code has been supplied as a Supplementary File.
