# Supplementary material for: How patch size and refuge availability change interaction strength and population dynamics: a combined individual- and population-based modeling experiment
Source: PeerJ. 2017 Feb 21;5:e2993. doi: 10.7717/peerj.2993 (PMC5322756; doi:10.7717/peerj.2993)
Supplement: Supplemental Information 2 — Contains the ODD and further information about parameters. [file peerj-05-2993-s002.pdf]

Supplemental Information for "How patch size and refuge availability change interaction strength and population dynamics: a combined individual- and population-based modeling experiment"

Yuanheng Li[1,2,3], Ulrich Brose[1,2], Katrin Meyer[4], and Björn C. Rall[1,2]

[1]German Centre for Integrative Biodiversity Research (iDiv) Halle-Jena-Leipzig, Deutscher Platz 5e, 04103 Leipzig, Germany
[2]Institute of Ecology, Friedrich Schiller University Jena, Dornburger-Str. 159, 07743 Jena, Germany
[3]Johann-Friedrich-Blumenbach Institute of Zoology and Anthropology, Georg-August-Universität Göttingen, Berliner Str. 28, 37073 Göttingen, Germany
[4]Department of Ecosystem Modelling, Georg-August-Universität Göttingen, Büsgenweg 4, 37077 Göttingen, Germany

1 ODD protocol for *in silico* feeding experiment

1.1 Overview

We modeled allometric predator-prey feeding interactions using an individual-based approach. The model description followed the ODD (Overview, Design concepts, Details) protocol (Grimm et al., 2006, 2010). The model was implemented in C++ using Code::Blocks as development environment.

1.2 Purpose

The purpose of the model is to estimate the dependencies of functional-response parameters on patch size and habitat complexity (represented by refuge availability) in a system with one predator and several prey items. As a preparation, we first investigated the maximum feeding rate without any explicit space properties, as we assumed that the maximum feeding rate is driven by physiological (mechanical) parameters such as chewing and digesting and does not scale with patch size or habitat complexity. Secondly, we assessed functional responses of predators to different prey densities in patches of different sizes and refuge availability.

1.3 Entities, state variables and scales

One predator and several prey individuals make up the agents of the model. The common state variables of predator and prey are individual identity, spatial coordinates (in cm), body mass (in mg), and body-mass dependent velocity. The predator is characterized by further state variables related to hunting and digestion. The parameter 'gut-fill' captures how many milligrams of food are currently in the gut of the predator, 'still-handling' captures how many time steps are still needed for handling prey, and 'prey-eaten' counts the number of prey items consumed by the predator.

The environment consists of a two-dimensional square area where its size and habitat complexity can be modified. To manipulate habitat complexity in the form of refuge availability for the prey, each cell in this area is characterized by the boolean state variable 'prey hiding'. This variable is set to 1 if prey individuals staying in this cell cannot be found by a predator, and to 0 otherwise. The predator and prey can move continuously in the area via random walk. Predator and prey are both able to enter all the cells, but the predator is not able

to find prey in refuge cells. We implemented non-periodic wall-like boundary conditions (Attard, 2006) to simulate a finite-sized patch.

In terms of scales, one grid cell is always 1 cm × 1 cm and the spatial extent of the patch is modified from 20 cm × 20 cm to 1000 cm × 1000 cm to account for different patch sizes. One time step represents one second and the simulations are ran for 3600 time steps corresponding to one hour.

1.4 Process overview and scheduling

The first process that is applied in this discrete-time model is prey movement (random walk with randomly chosen direction and allometric distance, i.e. the velocity of an individual scales with its body mass). The following processes are the predator's decisions and actions (Fig. 1 in the main text). First, the predator digests and afterwards if it has caught prey previously and is still handling it, the predator does the process of handling. Subsequently, if the predator is not handling prey anymore and its gut is full ('gut-fill' >= 60%), it rests, i.e. it does not take any actions in this time step. If the predator is not handling prey and is hungry ('gut-fill' < 60%), the predator moves according to the same rules as prey. After reaching the new position, the predator investigates if it encounters a prey in the current cell. If there is a prey individual in the same cell and this cell is not marked as refuge, the prey will be attacked. If the attack is successful, another prey item is placed randomly into the grid to keep prey density constant. The predator starts to handle the prey in the next time step.

1.5 Design concepts

Basic principles - This model mimics classical functional response experiments in the laboratory, but the model allows to explore much larger patch sizes than in a real laboratory arena. Moreover, the model can more easily be used to determine maximum feeding rates than laboratory experiments, because the amount of prey can be held constantly until maximum feeding rates are reached. *Emergence* - Functional responses are the main emerging pattern from the model, arising from the predator's efficiency at catching prey in patches of different sizes and habitat complexity (refuge availability). *Sensing* - Predator and prey are able to detect each other when they meet in the same cell which is not marked as refuge. Both predator and prey are able to detect patch edges and stop nearby when they reach an patch edge. Next time when this individual needs to move, it just moves according to the same rules as before. *Interaction* - The predator interact with prey by feeding on the prey when they meet on the same cell and the prey is not hidden. When the prey is in a refuge cell, the predator can enter that cell, but does not interact with the prey. *Stochasticity* - Random numbers are used in initialization of most variables, including coordinates of agents, the refuge availability of cells and the state variable 'gut-fill' of the predator. Stochasticity is also involved in the moving direction of agents when random walk applies. Bernoulli-distributed random numbers are drawn to determine the handling time for a prey item as time is a discrete variable in this model. *Observation* - In each *in silico* experiment, the number of prey items eaten by the predator is recorded at each time step. At the end of each simulation run, patch size, refuge availability (percentage of refuge cells), initial prey number, and 'prey-eaten' are recorded.

1.6 Initialization

Random values are used to initialize the spatial coordinates of all agents and choose refuge cells. All other initial parameters are listed in Table S1.

Table S1: Agents' state variables and parameters

sort	variables	unit	note
	gut-fill	rate	randomly initialized
state variable	still-handling	s	initially set to zero
	individual identify	number	
	prey-eaten	number	initially set to zero
	velocity	$\mathrm{cm\,s^{-1}}$	
	rate of successful attack	rate	
calculated parameter	full gut	mg	allometrically calculated
	digestion rate	rate	
	handling time	s	

Most species traits regulating the processes described above are calculated by allometric rules. These include velocities of predator and prey, V $[\,\mathrm{cm\,s^{-1}}\,]$, Eq. (S1a), (Peters, 1983), and the predator-specific traits: gut size, G $[\,\mathrm{mg}\,]$, Eq. (S1b), (Ibarrola et al., 2012); digestion rate, D $[\,\mathrm{mg\,s^{-1}}\,]$, Eq. (S1c), (Ibarrola et al., 2012); handling time, T_h $[\,\mathrm{s}\,]$, Eq. (S1d), (modified from Rall et al., 2012); and rate of successful attack, S_a unitless, Eq. (S1e), (Wahlström et al., 2000; data from Gergs and Ratte, 2009, and Gergs, 2011):

$$V = v_0\, M^{a_v} \tag{S1a}$$

$$G = g_0\, M^{a_g} \tag{S1b}$$

$$D = d_0\, M^{a_d} \tag{S1c}$$

$$T_h = h_0\, M_p^{a_{h,p}}\, M_n^{a_{h,n}} \tag{S1d}$$

$$S_a = a_0 \left(\frac{R}{R_{opt}}\, e^{1-\frac{R}{R_{opt}}} \right)^{\lambda} \tag{S1e}$$

where v_0, g_0 and d_0 are constants, a_v, a_g, and a_d are the allometric exponents, and M is the body mass of the corresponding individual. Subscripts $_p$ and $_n$ of M indicate predator and prey, respectively. As only few relevant studies were found on digestion mechanisms, we used generalized Rickers function (Persson et al., 1998; Persson and Brönmark, 2002b,a; Wahlström et al., 2000; Brose et al., 2008; Rall et al., 2011) to describe the scaling of attack success (S_a) depending on body size. This function consists of the maximum attack success a_0, predator-prey body-mass ratio, R and its optimum R_{opt} and a shaping parameter, λ. Predator and prey also possess some state variables to assist their decision making and activities, i.e. 'position' for all individuals; 'gut fullness' and 'still handling' for the predator; 'prey identity' for prey.

1.7 Submodels

1. Prey move. This process is the first one for each time step. Prey individuals do random walks consecutively according to their identity number. A random direction is generated (a double precision floating number from 0 to 2π) and position changes vertically and

horizontally are calculated according to prey velocity. Before updating the actual coordinates, wall boundary conditions are considered, checking if values of the coordinates would be beyond the boundaries. If so, the value is set close to the coordinate value of that edge but with a distance to the edge of 10^{-6} cm.

2. Digest. The state variable 'gut-fill' of the predator is subtracted by 'digestion rate' in this process. It is executed each time step even when the gut is already empty. If the value of gut fill drops below zero, it is set to zero.

3. Handle prey. This process is executed under the condition that the state variable 'still-handling' has a positive value. The value of 'still-handling' is reduced by one in this process.

4. Move. The predator does a random walk. If the predator is satisfied, meaning 'gut-fill' exceeds 0.6, this process is skipped.

5. Encounter and attack? Here, the coordinate of the predator is checked only when it is about to hunt ('gut-fill' $<$ 0.6). If the predator is currently in a refuge cell, the hunting process is forfeited. If it is not forfeited, the following actions are executed. 1) Check potential prey, checking if there exists one prey item that is in the same cell as the predator. Checking order follows the prey's identity numbers. As soon as one prey fulfills the condition, the checking is finished. 2) If there is a potential prey item, a random number (ranging from 0 to 1) is generated and compared to 'rate of successful attack' to decide if this prey flees.

6. Attack success? If the attacked prey does not flee (attack success), values of 'gut-fill' and 'still-handling' will be increased by the amounts calculated from the prey mass and 'prey-eaten' will be increased by one. As time is discrete in our model, a Bernoulli-distributed random number is drawn to make sure that the value of 'still-handling' is an integer and on average still satisfying the calculated handling time. If the prey flees (attack unsuccessful),

this time step ends.

7. Replace caught prey. If attack succeeds, the killed prey item (i.e. its identity number) would be randomly given a new set of spatial coordinates, but body mass never changes.

8. Output data. Data are recorded immediately after each of 3600 time steps. The number of prey eaten and relevant input values are recorded, i.e. body masses of agents, patch size, percentage of refuge cells and initial number of prey items.

2 Allometric handling time

We used data from Rall et al. (2012) to parameterize the equation for handling time (T_h, Eq. (2d)). We only selected the data for predation (parasitism excluded) and for short experimental duration ($\leq$ 10 minutes) to make the analysis. We fitted 67 data points to a linear mixed-effects model ('lme' in the package 'nlme' in R, Pinheiro et al. (2016); R Core Team (2016)). To correct for differences between studies, study identity was used as a random factor, and all the variables (explanatory variables, body masses of predator and prey species and dependent variable handling time) were ln-transformed. The statistics showed that the handling time increased with increasing prey mass and decreased with increasing prey mass and decreases with increasing predator mass (Fig. S1, Table S2).

Table S2: Statistics for handling time [a]

	Estimate	S.E.	p-value
intercept	3.624	0.839	0.0001
ln.pred [b]	-0.330	0.059	<0.0001
ln.prey [c]	0.173	0.051	0.0013

[a]handling time is ln-transformed
[b]ln-transformed predator body mass
[c]ln-transformed prey body mass

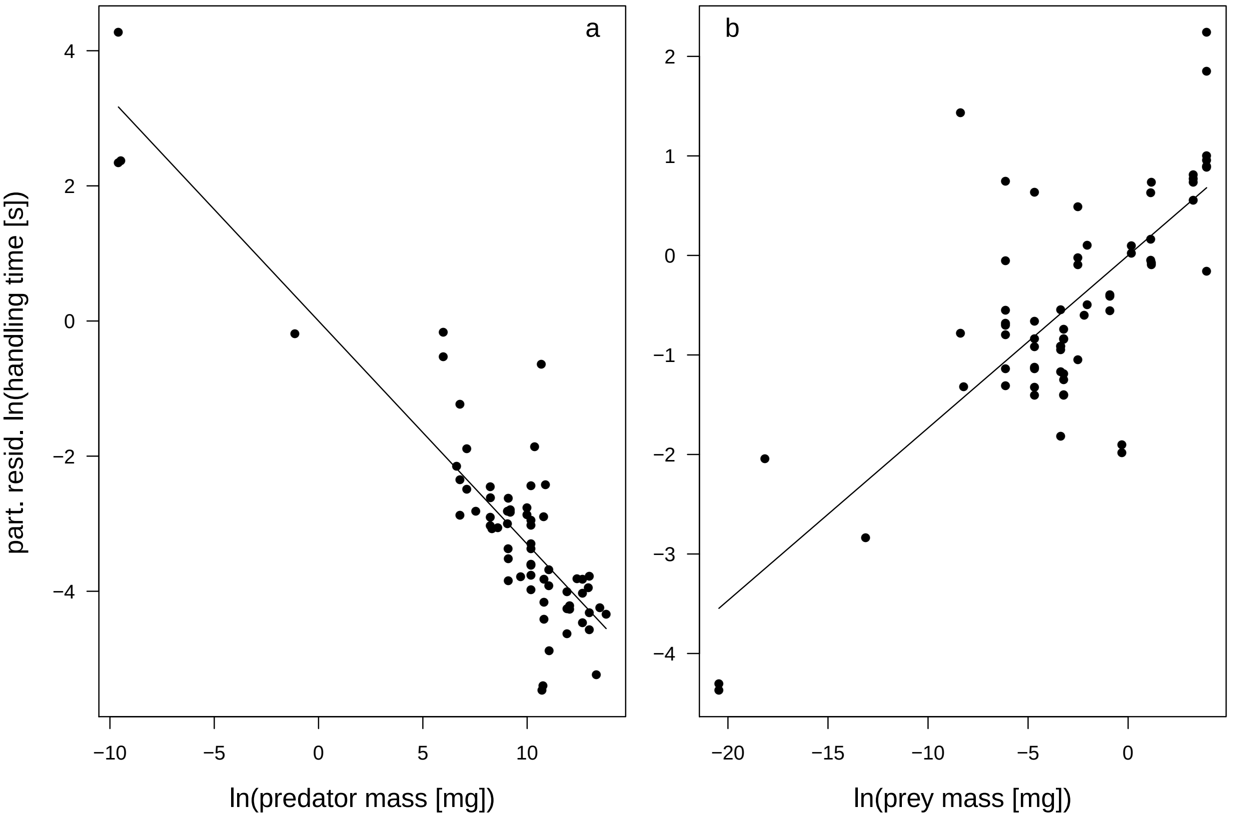

Figure S1: Statistical results for handling time (T_h) show that handling time decreases with increasing predator body mass and increases with increasing prey body mass. Data are ln-transformed before fitting. The partial residual of handling time is used as y-axis.

3 Preliminary model selection

We listed the statistical results of 'preliminary tests' (section "Functional response fitting" in the main text) on the types of scaling that functional-response parameters had with patch size or refuge availability, Tab. S3. For the simulations on predator-prey body-mass ratio of 100 and 200, the selected best model (according to the BIC) includes (1) half saturation density scaling with power law to patch size and exponentially to refuge availability; (2) Hill exponent scaling with power law to both patch size and refuge availability. For the simulation on body-mass ratio of 50, (1) the model is selected for the same scaling relationships of half saturation density as for body-mass ratio of 100 and 200; thus, (2) Hill exponent scales

exponentially with both patch size and refuge availability.

Table S3: Full model selection on scaling rules of functional-response parameters

model scaling rules [a][b]	BIC		
	100	200	50 [c]
eeee	45061.65	5710.646	3930.403
eepe [d]	45083.81	5709.091	3933.047
peee	45026.84	5709.33	3928.032 [e]
eeep	45057.89	5706.494	3930.711
epee	45219.88	5741.362	3940.024
pepe	45025.2	5707.415	3930.593
eepp	45091.41	5705.066	3932.338
eppe	45218.91	5754.17	3944.321
peep	45025.39	5706.341	3928.081
ppee	45228.25	5735.171	3938.638
epep	45231.84	5734.309	3940.953
pepp	45023.57	5703.671	3930.069
pppe	45185.07	5733.498	3941.549
eppp	45223.9	5731.754	3944.204
ppep	45236.62	5737.974	3944.041
pppp	45189.86	5729.644	3941.185

[a]scaling of N_0 to A, N_0 to R, h to A and h to R, subsequently
[b]N_0 half saturation density, h Hill exponent, A patch size, R refuge availability
[c]predator-prey body-mass ratio
[d]e/p: exponential/power law scaling
[e]lowest BIC value

4 *In silico* feeding experiments on other body-mass ratios

We additionally did functional response simulations for predator mass of 200 mg and 50 mg (prey mass of 1 mg). However, we reduced the numbers of patch sizes and refuge availabilities. The simulated patch sizes for both predator masses are $0.04\,m^2$, $0.64\,m^2$, $2.56\,m^2$, $16\,m^2$, $49\,m^2$ and $64\,m^2$; and the simulated refuge availabilities are 5% to 65%, in steps of 15%. All the following statistics follow the descriptions in the paper.

Table S4: Statistical results of functional-response parameters simulated on other body-mass ratios

predator mass	parameter [a]	estimate	s.e.	p-value
200	f_{max}	10.56	0.04	$< 2 \times 10^{-16}$
	b_{N_0}	1.504	0.159	$< 2 \times 10^{-16}$
	$ln(C_{N_0})$	5.338	0.067	$< 2 \times 10^{-16}$
	$ln(C_h)$	0.25	0.028	$< 2 \times 10^{-16}$
50	f_{max}	4.12	0.07	$< 2 \times 10^{-16}$
	b_{N_0}	1.423	0.257	3×10^{-8}
	$ln(C_{N_0})$	4.503	0.110	$< 2 \times 10^{-16}$
	$ln(C_h)$	0.26	0.047	3×10^{-8}

ht

[a]refer to Eq. (5) in the main text

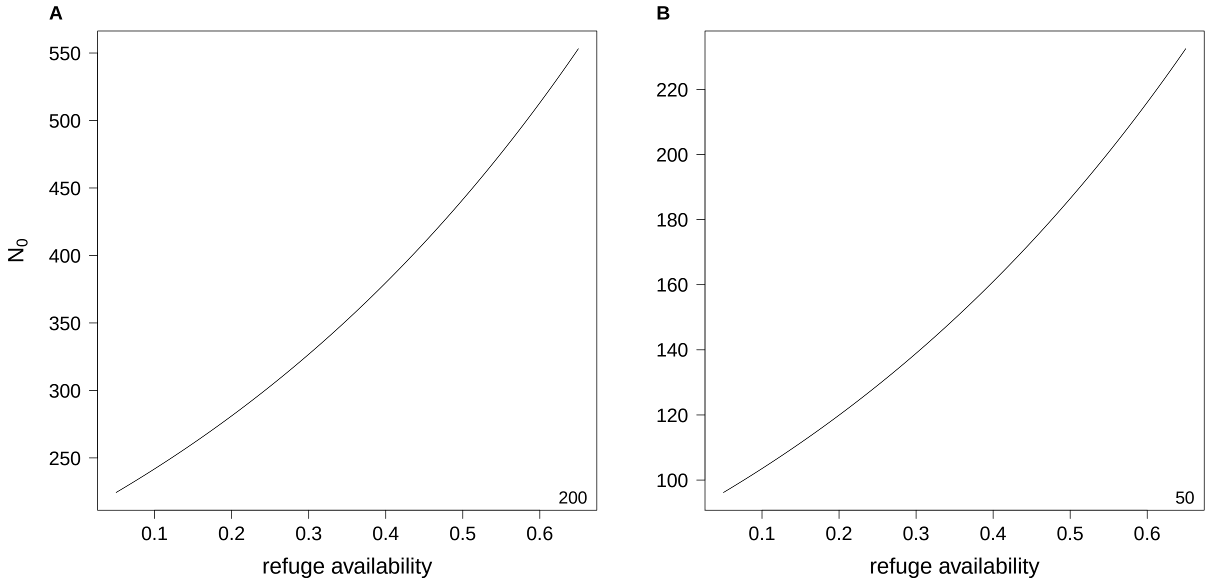

Figure S2: Effect of habitat complexity (expressed as refuge availability) on half saturation density. Panel A and B show the results for body-mass ratio of 200 and 50, respectively (right-bottom corner).

The maximum feeding rate, f_{max}, were 4.12 and 10.56 individuals for predator mass of 50 mg and 200 mg (Tab. S4). The final models for predator mass of 50 mg and 200 mg based on the BIC selection showed the same dependencies, including an exponential scaling of half saturation density with refuge availability but no scaling with patch size (refer to Eq. (5a)).

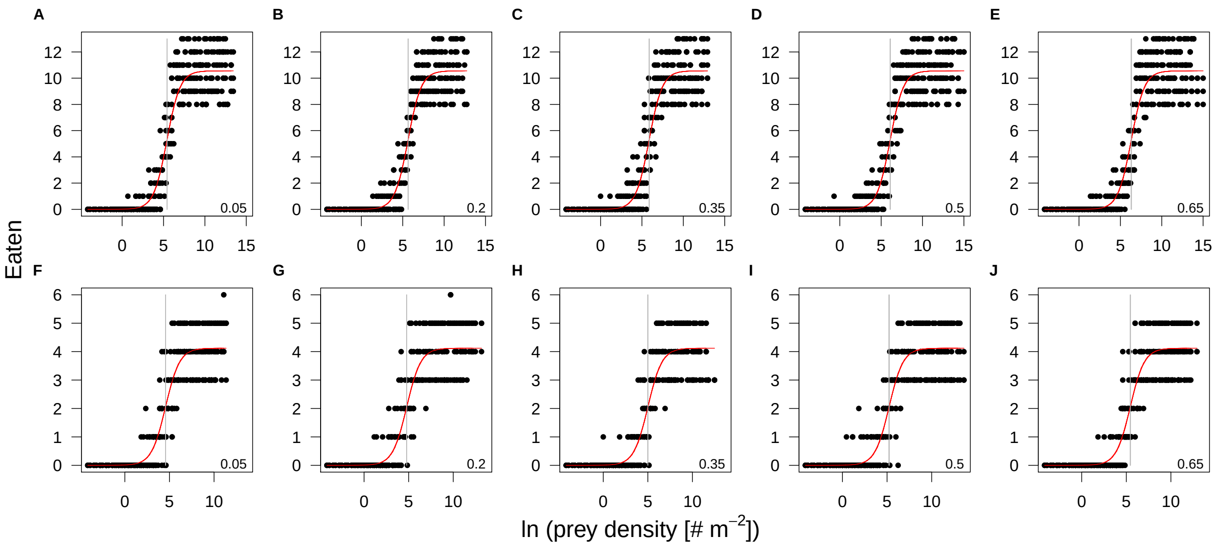

Figure S3: Results of *in silico* functional response experiments (black dots) and their corresponding fits (red lines). The panels are arranged by increasing refuge availability and increasing body-mass ratios of predator-prey. The upper row (panels A to E) are for body-mass ratio of 200 and lower (panels F to J) for 50. Each row starts with data of 5% refuge availability (A, uppermost left) to 65% (O, lowermost right). All prey densities are ln-transformed. The grey vertical lines denote the half saturation densities.

Consistent with the result for predator mass of 100 mg in the main text, they did not include any scaling of Hill exponent for both predator masses (refer to Eq. (5b)). The half saturation densities increased with increasing refuge availability, $b_{N_0} = 1.504$ for predator mass of 200 mg and $b_{N_0} = 1.423$ for predator mass of 50 mg , see Fig. S2 and Tab. S4. The ln-transformed intercepts of the half saturation density were 5.338 and 4.503 for predator mass of 200 mg and 50 mg, respectively (Tab. S4). The estimated Hill exponents across all patch sizes and refuge availabilities were 1.284 for predator mass of 200 mg and 1.300 for predator mass of 50 mg (Tab. S4). The fitted functional response curves are of the same shape but feedings are realized at higher densities with increasing refuge availabilities (Fig. S3).

Reference

Attard, P. (2006). Non-periodic boundary conditions for molecular simulations of condensed matter. *Molecular Physics*, 104(12):1951–1960.

Brose, U., Ehnes, R. B., Rall, B. C., Vucic-Pestic, O., Berlow, E. L., and Scheu, S. (2008). Foraging theory predicts predatorprey energy fluxes. *Journal of Animal Ecology*, 77(5):1072–1078.

Gergs, A. (2011). *Modelling foraging behaviour in the insect predator Notonecta maculata using the individuals approach / vorgelegt von: Andr Gergs*. PhD thesis, RWTH Aachen University.

Gergs, A. and Ratte, H. T. (2009). Predicting functional response and size selectivity of juvenile Notonecta maculata foraging on Daphnia magna. *Ecological Modelling*, 220(23):3331–3341.

Grimm, V., Berger, U., Bastiansen, F., Eliassen, S., Ginot, V., Giske, J., Goss-Custard, J., Grand, T., Heinz, S. K., Huse, G., Huth, A., Jepsen, J. U., Jrgensen, C., Mooij, W. M., Mller, B., Peer, G., Piou, C., Railsback, S. F., Robbins, A. M., Robbins, M. M., Rossmanith, E., Rger, N., Strand, E., Souissi, S., Stillman, R. A., Vab, R., Visser, U., and DeAngelis, D. L. (2006). A standard protocol for describing individual-based and agent-based models. *Ecological Modelling*, 198(1-2):115–126.

Grimm, V., Berger, U., DeAngelis, D. L., Polhill, J. G., Giske, J., and Railsback, S. F. (2010). The ODD protocol: A review and first update. *Ecological Modelling*, 221(23):2760–2768.

Ibarrola, I., Arambalza, U., Navarro, J. M., Urrutia, M. B., and Navarro, E. (2012). Allomet-

ric relationships in feeding and digestion in the Chilean mytilids Mytilus chilensis (Hupé), Choromytilus chorus (Molina) and Aulacomya ater (Molina): A comparative study. *Journal of Experimental Marine Biology and Ecology*, 426-427:18–27.

Persson, A. and Brönmark, C. (2002a). Foraging capacities and effects of competitive release on ontogenetic diet shift in bream, *Abramis brama*. *Oikos*, 97(2):271–281.

Persson, A. and Brönmark, C. (2002b). Foraging capacity and resource synchronization in an ontogenetic diet switcher, Pikeperch (*Stizostedion lucioperca*). *Ecology*, 83(11):3014–3022.

Persson, L., Leonardsson, K., de Roos, A. M., Gyllenberg, M., and Christensen, B. (1998). Ontogenetic scaling of foraging rates and the dynamics of a size-structured consumer-resource model. *Theoretical Population Biology*, 54(3):270–293.

Peters, R. H. (1983). *The ecological implications of body size*. Number 2 in Cambridge studies in ecology. Cambridge University Press, New York, repr edition.

Pinheiro, J., Bates, D., DebRoy, S., Sarkar, D., and R Core Team (2016). *nlme: Linear and Nonlinear Mixed Effects Models*. R package version 3.1-123.

R Core Team (2016). *R: A Language and Environment for Statistical Computing*. R Foundation for Statistical Computing, Vienna, Austria.

Rall, B. C., Brose, U., Hartvig, M., Kalinkat, G., Schwarzmuller, F., Vucic-Pestic, O., and Petchey, O. L. (2012). Universal temperature and body-mass scaling of feeding rates. *Philosophical Transactions of the Royal Society B: Biological Sciences*, 367(1605):2923–2934.

Rall, B. C., Kalinkat, G., Ott, D., Vucic-Pestic, O., and Brose, U. (2011). Taxonomic versus allometric constraints on non-linear interaction strengths. *Oikos*, 120(4):483–492.

Wahlström, E., Persson, L., Diehl, S., and Byström, P. (2000). Size-dependent foraging efficiency, cannibalism and zooplankton community structure. *Oecologia*, 123(1):138–148.
